# *Pseudomonas aeruginosa* PilY1 Binds Integrin in an RGD- and Calcium-Dependent Manner

**DOI:** 10.1371/journal.pone.0029629

**Published:** 2011-12-29

**Authors:** Michael D. L. Johnson, Christopher K. Garrett, Jennifer E. Bond, Kimberly A. Coggan, Matthew C. Wolfgang, Matthew R. Redinbo

**Affiliations:** 1 Department of Biochemistry and Biophysics, University of North Carolina at Chapel Hill, Chapel Hill, North Carolina, United States of America; 2 Division of Plastic and Reconstructive Surgery, Department of Surgery, Duke University Medical Center, Durham, North Carolina, United States of America; 3 Department of Microbiology and Immunology, University of North Carolina at Chapel Hill, Chapel Hill, North Carolina, United States of America; 4 Cystic Fibrosis/Pulmonary Research and Treatment Center, University of North Carolina at Chapel Hill, Chapel Hill, North Carolina, United States of America; 5 Department of Chemistry, University of North Carolina at Chapel Hill, Chapel Hill, North Carolina, United States of America; University of Osnabrueck, Germany

## Abstract

PilY1 is a type IV pilus (tfp)-associated protein from the opportunistic pathogen *Pseudomonas aeruginosa* that shares functional similarity with related proteins in infectious *Neisseria* and *Kingella* species. Previous data have shown that PilY1 acts as a calcium-dependent pilus biogenesis factor necessary for twitching motility with a specific calcium binding site located at amino acids 850–859 in the 1,163 residue protein. In addition to motility, PilY1 is also thought to play an important role in the adhesion of *P. aeruginosa* tfp to host epithelial cells. Here, we show that PilY1 contains an integrin binding arginine-glycine-aspartic acid (RGD) motif located at residues 619–621 in the PilY1 from the PAK strain of *P. aeruginosa*; this motif is conserved in the PilY1s from the other *P. aeruginosa* strains of known sequence. We demonstrate that purified PilY1 binds integrin *in vitro* in an RGD-dependent manner. Furthermore, we identify a second calcium binding site (amino acids 600–608) located ten residues upstream of the RGD. Eliminating calcium binding from this site using a D608A mutation abolished integrin binding; in contrast, a calcium binding mimic (D608K) preserved integrin binding. Finally, we show that the previously established PilY1 calcium binding site at 851–859 also impacts the protein's association with integrin. Taken together, these data indicate that PilY1 binds to integrin in an RGD- and calcium-dependent manner *in vitro*. As such, *P. aeruginosa* may employ these interactions to mediate host epithelial cell binding *in vivo*.

## Introduction

*Pseudomonas aeruginosa* is a Gram-negative, opportunistic pathogen prevalent in immunocompromised patients, burn victims, and people with cystic fibrosis. *P. aeruginosa* can infect any part of the body, but typically targets the respiratory tissues, skin abrasions, and the urinary tract [1]. This pathogen accounted for ∼11% of the hospital-acquired infections reported in the U.S in 2002 [2], [3], and has also been shown to infect other mammalian species, as well as insects and plants [4], [5]. *P. aeruginosa* uses a range of methods to resist the effects of antibiotics, including efflux pumps, adaptive mutagenesis, and protective biofilms [6], [7], [8]. As such, *Pseudomonas aeruginosa* presents a significant challenge to human health.

*P. aeruginosa* employs type IV pili (tfp) for twitching motility and infection. The precise mechanism of host cell attachment has remained unclear, although evidence exists that loops of the major pilus structural subunit PilA exposed at the tip of the pilus fiber bind to gangliosides GM1 and GM2 [9]. In contrast, some data indicate that several *P. aeruginosa* clinical isolates, as well as laboratory strains, do not employ GM1 and GM2 during host cell attachment [10], [11]. As such, it has been proposed that other factors on the *Pseudomonas* tfp are involved in binding to target cells [12], [13].

Recent studies have suggested that host cell integrin proteins play a role in *Pseudomonas* infection. Anti-integrin antibodies were shown to reduce *P. aeruginosa* attachment to host cells [14]. Specifically, antibodies to the αVβ5 integrin and αVβ3 integrin were effective at disrupting *P. aeruginosa* binding to host cells, with antibodies to αVβ5 integrin having the most pronounced effect [14]. Integrins are present on the epithelial cell surface of tissues infected by *P. aeruginosa*; indeed, αVβ5 integrin is highly expressed in the lungs [15]. The presence of *P. aeruginosa* has also been shown to increase the expression of integrin subunits αV, α5, and β1 in epithelial cells [16], [17]. Integrins and integrin-like proteins are found widely in animals, plants, and insects, all targets of *P. aeruginosa* infection [18]. Integrins are responsible for a range of cellular processes, including cell-cell attachment, cellular signaling, and angiogenesis [19]. The most common ligands for integrins are proteins that contain an arginine-glycine-aspartic acid (RGD) sequence, although other short peptides have also been found to mediate integrin-protein interactions (*e.g.*, leucine-aspartic acid-valine or LDV).

The PilC class of proteins in *Neisseria gonorrhoeae*, *Neisseria meningitidis*, and *Kingella kingae* have been characterized as tfp biogenesis factors and as proteins involved in adhering to target tissues [20], [21], [22]. Generally, PilC N-terminal regions are associated with adhesion domains, while the C-terminal domains regulate tfp biogenesis. *P. aeruginosa* PilY1 shares sequence homology with the C-terminal regions of the PilCs proteins, and this domain in PilY1 has been shown to be a calcium-mediated tfp biogenesis factor (Table S1) [23]. Indeed, PilY1 is required for both twitching and swarming motility and adhesion to host cells [23], [24], [25]. The N-terminal domains of PilY1 and the PilCs share low sequence similarity, which might be expected if these domains are involved in target cell-specific activities. The target tissues of the *Neisseria* strains and *Kingella* are likely to be distinct from those targeted by *P. aeruginosa*. Here, we focus on putative regions of PilY1 involved in host cell attachment and we show that purified PilY1 binds to integrins in an RGD- and calcium-dependent manner *in vitro*.

## Results

### PilY1 contains a conserved integrin binding motif

**Figure 1:**
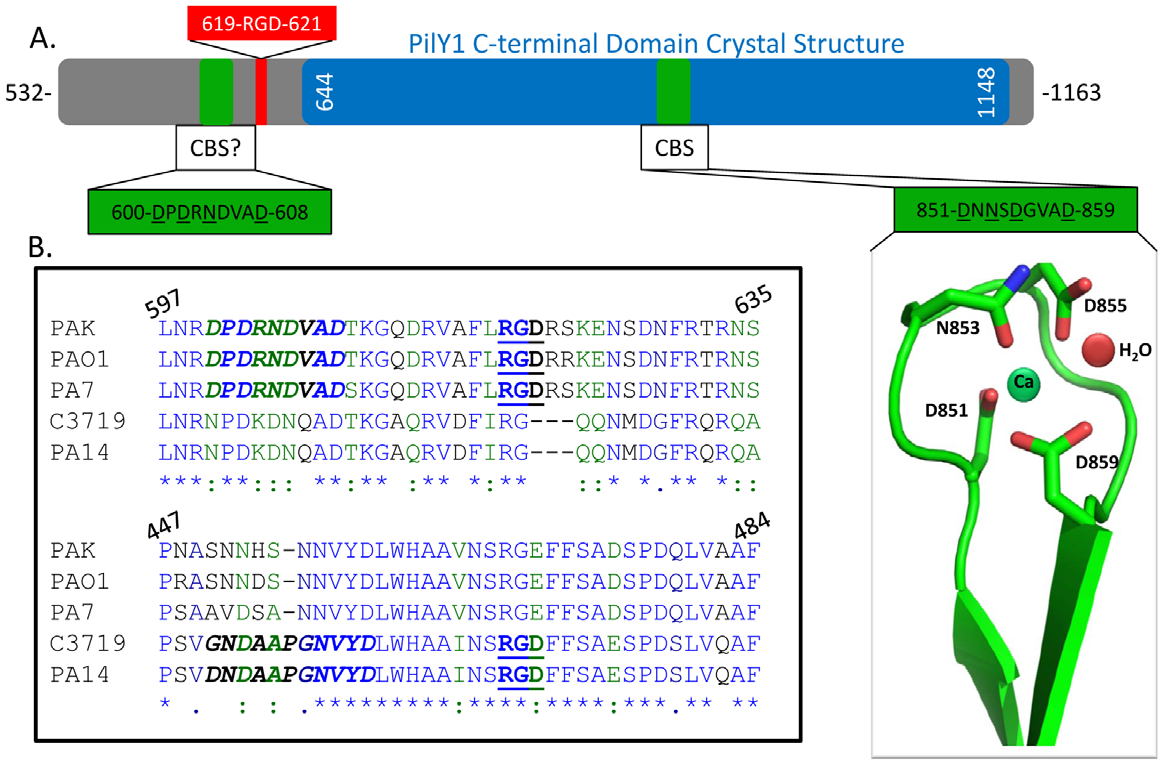
*P. aeruginosa* PilY1 strains contain conserved integrin binding residues RGD and conserved putative calcium binding site. (a) A bar representation of PilY1. The consensus c-terminal pilus biogenesis domain is in blue and the green represents the n-terminal addition to the previously examined construct. Calcium binding motifs are highlighted in yellow and the RGD is highlighted in orange. (b) Five varying strains of *P. aeruginosa* PilY1 were aligned using the biology workbench server [26], [27]. Blue residues and an “*” corresponds to identical residues throughout the 5 strains, green residues and a “:” correspond to highly conserved residues, and navy residues and a “.” corresponds to mildly conserved residues (e.g. alanine and leucine). Reference numbers are for PAK_287 PilY1 (the strain used in this study).

Given the importance of the tfp associated PilY1 protein in host tissue adherence and the ability of antibodies targeting the RGD binding domains of αVβ5 and αVβ3 integrins to reduce interactions between *P. aeruginosa* and host cells, we examined PilY1's sequence to locate possible integrin binding motifs (RGD or LDV) [14], [25]. PilY1 was found to contain a conserved RGD motif (Figure 1A, 1B). No other type IV pilus proteins or known surface proteins in *P. aeruginosa* contained an RGD or other putative integrin binding sequence [26], [27]. One RGD motif was found in each of the PilY1s of *P. aeruginosa* strains of known sequence. For example, in the PilY1 from *P. aeruginosa strain K* (PAK), the RGD is located at residues 619–621; however, in the PAO1 *P. aeruginosa* strain PilY1, the RGD is at 657–659 (Figure 1B). Based on these observations, we hypothesized that PilY1 binds to integrin in an RGD-dependent manner.

### PilY1 binds integrin in an RGD-dependent manner

**Figure 2:**
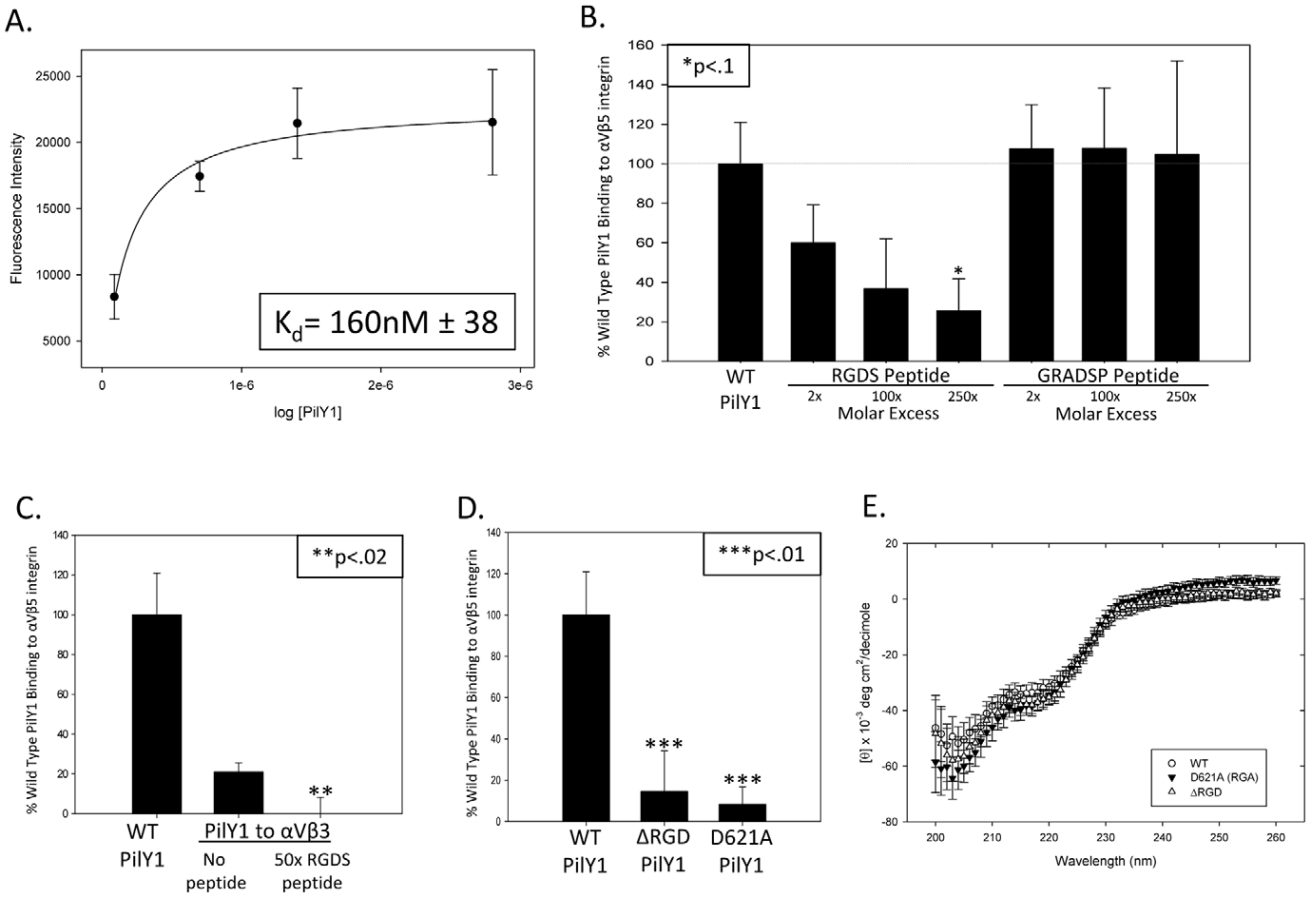
Purified PilY1 binds to integrin in an RGD dependent manner. (a) PilY1 was titrated onto a solid phase binding assay keeping the concentration of αVβ5 integrin constant at 2 µg/mL. The curve was fit to the ligand binding, one site saturation equation f = Bmax*abs(x)/(K_d_+abs(x)) with an r^2^ = 0.98. The K_d_ was calculated at 164 nM±38. (b–d) Wild type PilY1 was established as a reference point and the value all measurements were compared to. (b) RGDS inhibitor peptide or GRADSP control peptide were added in 2, 100, and 250 fold molar excess to PilY1. “*” corresponds to p<.1 compared to PilY1 binding to αVβ5 integrin. (c) PilY1 was added with or without 50 fold molar excess of the inhibitor RGDS peptide to 2 µg/mL of αVβ3 integrin. “**” corresponds to p<.02 compared to PilY1 binding to αVβ3 integrin. (d) ΔRGD (Δ619–621) or D621A mutations of PilY1 were added to 2 µg/mL of αVβ5 integrin. “***” corresponds to p<.01 compared to PilY1 binding to αVβ5 integrin. (e) Molar ellipticity values were calculated for the respective wavelength scans and compared.

To test the hypothesis that PilY1's RGD motif mediates contacts with integrins, we created a purified form of PilY1 that starts at amino acid 532 and extends to C-terminus of the protein (residue 1163) and tested the ability of 532–1163 PilY1 to bind to αVβ5 using an integrin binding assay. Titration of increasing concentrations of 532–1163 PilY1 showed that mid-nanomolar concentrations of this purified protein binds specifically to αVβ5 (Figure 2A). Next, we employed synthetic RGDS and GRADSP peptides to compete with PilY1 binding to αVβ5 integrin. We found that that the RGDS peptide significantly reduced PilY1 binding to αVβ5 integrin (p<0.10), while the GRADSP control peptide had no effect (Figure 2B). Because previous reports also indicated that PAK binding to host cells can be reduced by using an anti-αVβ3 integrin antibody, we also examined PilY1 binding to αVβ3. We found that purified 532–1163 PilY1 binds to αVβ3 integrin in an RGD-dependent manner, although with a lower apparent affinity than observed with αVβ5 integrin (Figure 2C) [14]. We therefore used αVβ5 as the primary integrin of this study. Finally, we tested two RGD mutants of 532–1163 PilY1, D621A (RGA) and Δ619–621 (ΔRGD), for their ability to bind to αVβ5 integrin *in vitro*. Deleting the RGD residues or mutating the aspartic acid residue at position 621 to alanine significantly reduced the association of PilY1 with αVβ5 integrin (Figure 2D). Importantly, these mutations did not alter the global secondary structure or the melting temperature of purified 532–1163 PilY1 (Figure 2E, Table S2). Taken together, these data indicate that *P. aeruginosa* PilY1 binds to integrins *in vitro* in an RGD-dependent manner.

### PilY1 has a second calcium binding site

**Figure 3:**
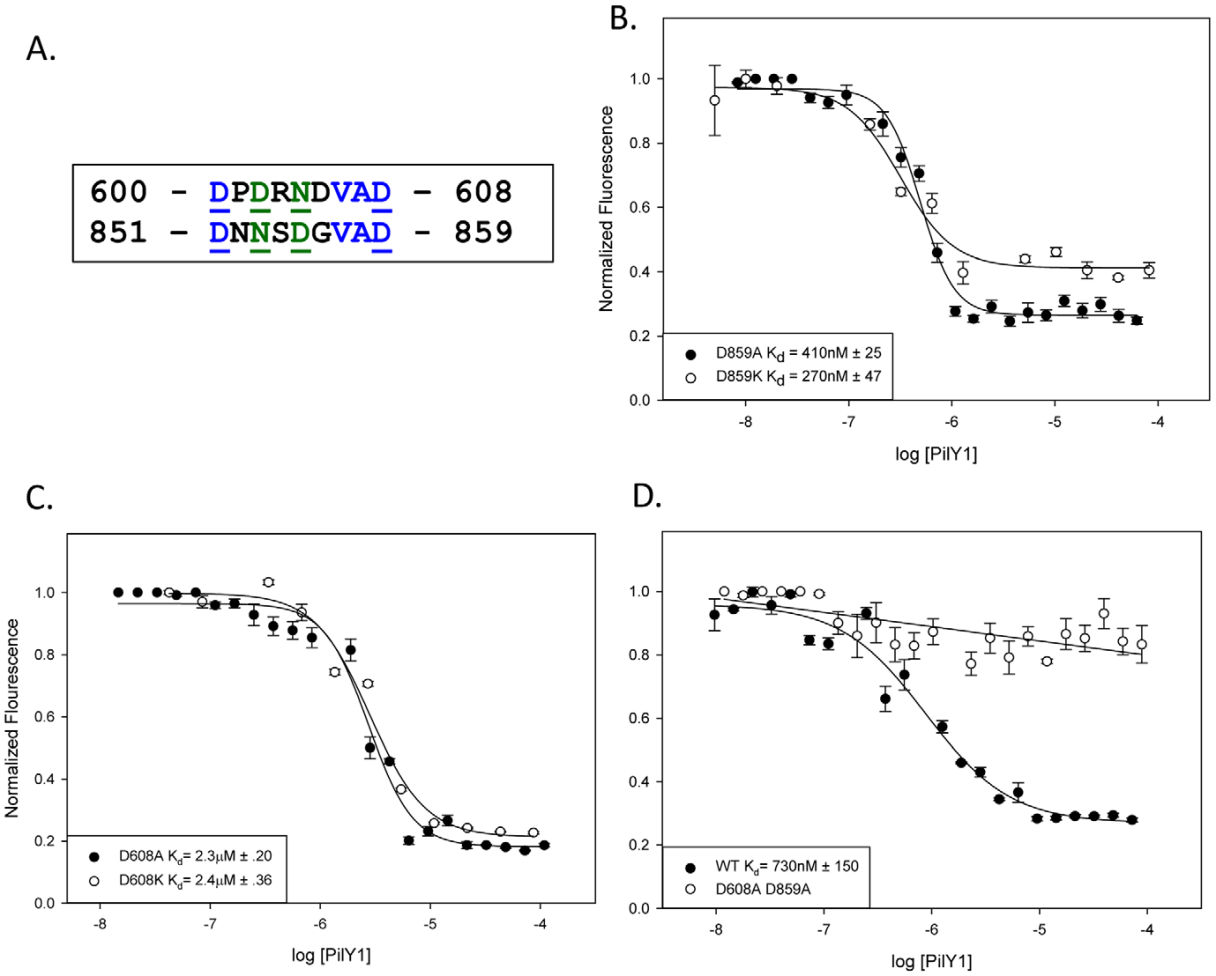
PilY1 has two functional calcium binding sites. (a) An alignment of the two calcium binding sites in PilY1 was performed as in figure 1. Calcium coordinating residues are underlined. (b, c, and d) A calcium competition binding assay using Oregon Green was performed with (b) D859A and D859K, (c) D608A and D608K, and (d) wild type and D608A/D859A. Binding curves were modeled to one-site competition (D859A, D859K, D608A, D608K, and wild type), or linear line (D608A/D859A). Error represents standard error of the mean.

A second potential calcium binding site was noted in the sequence of *P. aeruginosa* PilY1 between residues 600 and 608, which is in close proximity to the RGD motif (619–621) described above (Figure 1A). Recall that a calcium binding site at residues 851–859 in PilY1 was shown in previous work to be critical to tfp biogenesis and twitching motility [23]. The sequences of the two calcium binding sites are closely related (Figure 3A), and the putative 600–608 site is conserved in PilY1s of known sequence (Figure 1B). Indeed, in the *P. aeruginosa* PilY1 sequences examined, this second potential calcium binding site was always found 8–10 residues upstream of the conserved RGD motif (Figure 1B). To test the 600–608 site's ability to bind calcium in purified 532–1163 PilY1, we eliminated the previously published calcium binding site using either a D859A or D859K mutation [23], and measured calcium binding. The D859A and D859K forms of 532–1163 PilY1 exhibited K_d_'s for calcium binding of 412 and 266 nM, respectively (Figure 3B). Thus, the 600–608 site appeared to bind calcium. We next created corresponding mutations (D608A, D608K) in the 532–1163 PilY1 construct, and found that these variants bound calcium with 2.3–2.4 µM affinity, similar to that reported previously for the 851–859 PilY1 site (Figure 3C) [23]. Finally, we created a D608A/D859A double-mutant form of 532–1163 PilY1 and compared its calcium binding to wild-type 532–1163 PilY1. We found that the double-mutant exhibited only non-specific calcium binding, while wild-type 532–1163 PilY1 associated with calcium with a 700 nM affinity (Figure 3D). The variant forms of 532–1163 PilY1 examined *in vitro* in these studies maintained an overall wild-type fold and melting temperature, indicating that these changes did not alter the stability of the proteins (Figure S1A–E; Table S2). Additionally, mutations in the RGD sequence adjacent to the 600–608 site did not alter calcium binding by these purified forms of 532–1163 PilY1 (Figure S2A). The 532–1163 PilY1 construct demonstrated 1.3 µM and 600 nM affinity binding to Zn and Mn, respectively, and non-specific binding to Mg (Figure S3A–D). However, due to PilY1's structural similarity to established calcium binding sites, we consider calcium to be the primary metal bound to PilY1 [23], [28], [29], [30]. Taken together, these observations support the conclusion that purified 532–1163 PilY1 contains two calcium binding sites, the previously characterized one at 851–859 and a newly identified one at 600–608 that is ten residues N-terminal to PilY1's RGD motif.

### PilY1 binds integrin in a calcium dependent manner

**Figure 4:**
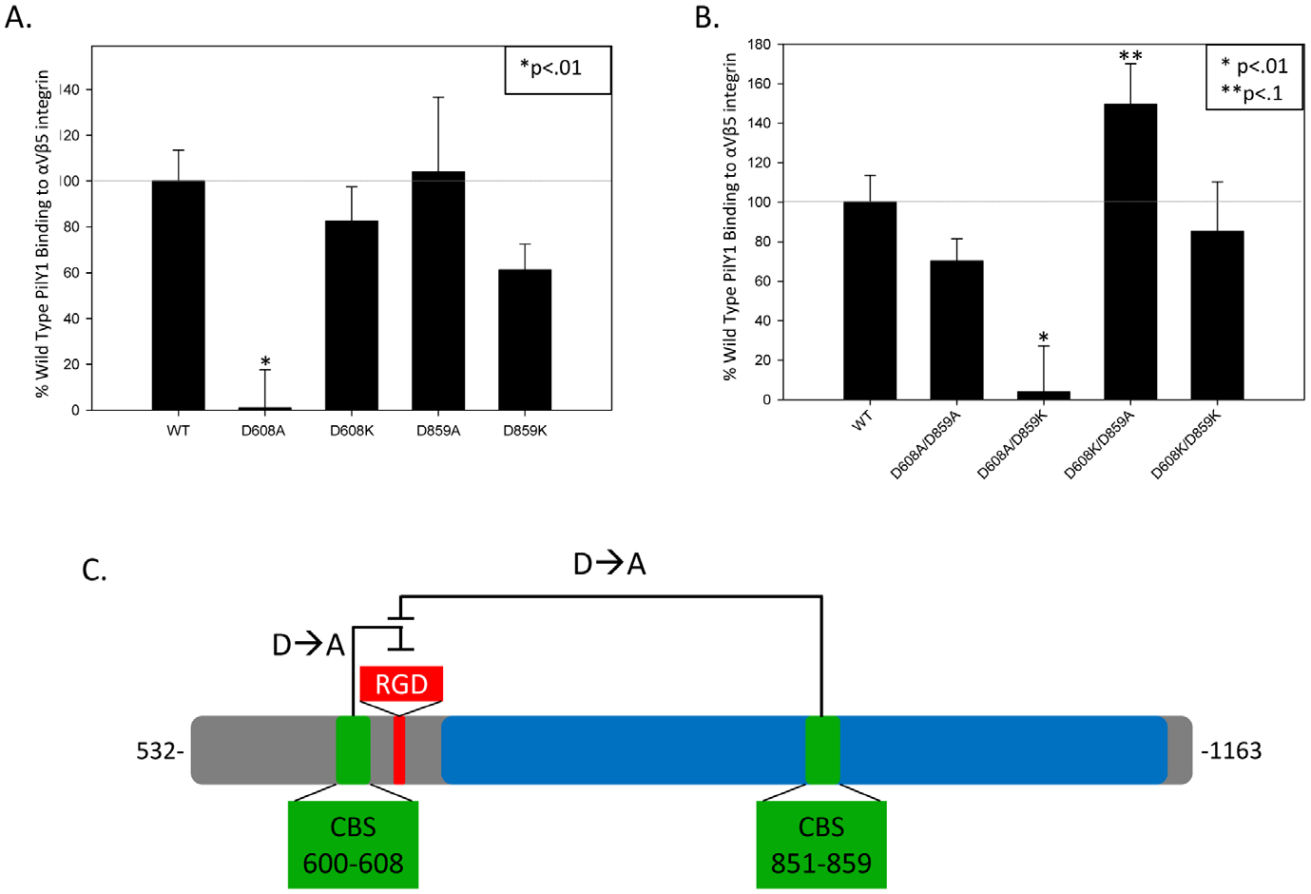
Calcium binding sites play a role in PilY1's ability binds to integrin. (a) D608A, D608K, D859A, D859K (b) D608A/D859A, D608A/D859K, D608K/D859A, and D608K/D859K mutations of PilY1 were added to αVβ5 integrin as seen in figure 2B. “*” corresponds to p<.01 compared to wild type PilY1 binding to αVβ5 integrin and “**” corresponds to p<.1 as compared to wild type PilY1 binding to αVβ5 integrin. (c) This model represents the calcium binding effects on PilY1 binding to integrin. D to A mutations represent mutations in the final aspartic acid of the calcium binding site.

Because the 600–608 calcium binding site in PilY1 is close in sequence to the RGD motif, we next sought to examine whether calcium binding impacted the association between 532–1163 PilY1 and integrin. We found that D859A, D859K, and D608K mutant forms of 532–1163 PilY1 exhibit wild-type or only slightly reduced binding to αVβ5; in contrast, the D608A mutant of purified PilY1 was significantly inhibited in its binding to this integrin (Figure 4A). Thus, eliminating the charged D608 side chain proximal to the RGD motif impacted PilY1's interaction with αVβ5. We then examined double mutant forms of 532–1163 PilY1 and found that the two calcium binding sites interacted functionally with respect to integrin binding (Figure 4B). For example, the D608A/D859A variant of 532–1163 PilY1 shows nearly wild-type levels of αVβ5 binding (Figure 4B); recall that the D608A form of PilY1 exhibited little integrin binding *in vitro* (Figure 4A). Thus, D859A appears to act as a dominant positive over the D608A mutation. Similarly, the D608K/D859A PilY1 shows increased integrin binding relatively to wild-type (Figure 4B). In contrast, adding the D859K mutation to either D608A or D608K in PilY1 had no impact on integrin binding relative to the 608 mutants alone (Figure 4B). Taken together, these data suggest that both calcium binding sites in PilY1 impact integrin binding *in vitro*. Note that the single- and double-mutants considered here did not bind calcium (Figure S2B), and exhibited wild-type CD spectra and melting temperatures (Figure S1A–E, Table S2), indicating that the overall structure of these purified proteins was not altered.

The crystal structure of the C-terminal domain of PilY1 does not contain the 600–621 region; as such, it is not known whether the two calcium binding sites are in close structural proximity. However, it is possible that they interact physically, and that the functional impact of one upon the other is via the swapping of an aspartic acid from one site (*e.g.*, D851 or D855) into the other site (600–608). To test this hypothesis, we created two triple mutant forms of 532–1163 PilY1, D608A/D851A/D859A and D608A/D855A/D859A, and one quadruple mutant form, D608A/D851A/D855A/D859A. Both triple mutants and the quadruple mutant show wild type levels of binding (Figure S4). Thus, we conclude that an interaction between the two calcium binding sites in PilY1 is not primarily associated with aspartic acids in one site helping to coordinate calcium in the other site. However, taken together, these data indicated that a functional interaction between the two calcium binding sites in PilY1 impacts the ability of the RGD motif in this protein to bind to integrins *in vitro*.

## Discussion

*P. aeruginosa* is an established and increasingly antibiotic resistant pathogen that predominantly infects patients with compromised defense mechanisms. Toward this end, we sought to understand how *P. aeruginosa* binds to host cells, a necessary first step in infection. We focused on the type IV pilus-associated protein PilY1, which is involved in bacterial motility and is related in sequence to factors in other bacteria required for host cell binding. We show that PilY1 contains an integrin binding RGD motif that mediates contact between purified PilY1 and integrin proteins *in vitro*. Additionally, we show that PilY1 contains two calcium binding sites and that each site impacts the protein's ability to associate with integrin (Figure 4C). Both the calcium binding sites and the RGD motif are conserved in all PilY1 sequences from *P. aeruginosa* strains reported to date (Figure 1B).

It was not anticipated that the PilY1 calcium binding sites, particularly the one distal (851–859) to the RGD sequence, would impact integrin binding. In fact, D859A can be seen as a dominant positive with respect to integrin binding (Figure 4C). For example, while the D608A mutant form of PilY1 is significantly reduced in integrin binding, combining it with D859A to create a D608A/D859A double-mutant produces a protein that is capable of binding to integrin (Figure 4B). Further, a D608K/D859A double-mutant exhibits higher integrin binding than either single mutant or the wild-type protein (Figure 4B). In spite of these *in vitro* data, however, the molecular contacts involved in these effects are not clear, in part because the region between residues 600 and 643 in PilY1 has not been elucidated structurally. It is tempting to speculate that a physical association exists between the two calcium binding sites that impacts RGD presentation for integrin binding. Such speculation is supported by the data outlined here, but remains speculation until future studies to examine the structure of this region of PilY1 are complete.

Integrins and integrin-like proteins are present in plants, insects, and the animal kingdom. They are up-regulated during cellular stress and recovery [31], [32], [33]; indeed, the presence of *P. aeruginosa* itself up-regulates the expression levels of the αV integrin subunit [16]. In addition, integrins are established targets for bacterial pathogens. *Bordetella pertussis* protein pertactin, containing two RGD motifs, has been shown to adhere to Chinese hamster ovary cells in an RGD-dependent manner [34], [35]. Pertactin is a member of the auto-transporter family, many of which contain RGD motifs mediating adherence to integrins [36]. However, there are conflicting data on whether auto-transporter RGDs are relevant in mouse models of infection [37]. Still, other *in vitro* and *in vivo* studies have shown that the following bacteria bind integrin in an RGD-dependent manner: *Pyrenophora tritici-repentis* (a wheat pathogen), *Mycoplasma conjunctivae* (sheep pathogen), and the mammalian pathogens *Klebsiella pneumoniae* and *Helicobacter pylori* [38], [39], [40]. Integrins are also known targets for viral pathogens. Proteins in hepatitis C virus, coxsackievirus A9, human herpes virus 8, Epstein-Barr virus, and adenovirus bind to host cells in an RGD dependent manner [41], [42], [43], [44], [45]. In summary, integrins have the potential to be important target proteins for PilY1-mediated attachment of *P. aeruginosa*.

**Figure 5:**
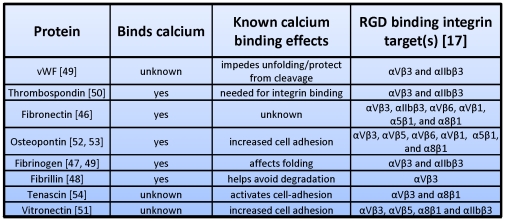
Calcium effects on RGD binding proteins.

Interestingly, calcium has also been shown to play a vital role in host RGD substrate:integrin binding events (Figure 5). Calcium binding helps in the folding of the blood clotting factor fibrinogen, and it protects against cleavage and degradation of the hemostasis protein von Wildenbrand factor and the elastin scaffold protein fibrillin (Figure 5) [46], [47], [48], [49]. Some integrin substrates, such as thrombospondin, use cooperative calcium binding at multiple sites to facilitate integrin binding, while calcium increases adhesion in tenascin, vitronectin, and osteopontin to target integrins (Figure 5) [50], [51], [52], [53], [54]. Thus, there is precedent for calcium regulation of RGD-mediated contacts with integrins.

PilY1 is an established pilus biogenesis factor [23] and has been implicated in *P. aeruginosa* adhesion to host cells by functional studies [25] and due to its homology to the PilCs adhesin proteins of *Neisseria* [55]. Here, we provide evidence that PilY1 serves as an adhesin that binds to integrins in an RGD-dependent manner. Furthermore, we show that integrin binding is regulated by two distinct calcium binding sites in PilY1. These data provide an *in vitro* advance in our understanding of PilY1 structure and function. It will be of interest to determine in future studies whether PilY1 and integrins are involved in *P. aeruginosa* attachment to host cells *in vivo*.

## Methods

### PilY1 constructs and protein purification

Site-directed mutagenesis was performed to produce D608A, D608K, D621A, Δ619–621, D859A, D859K, D608A/D859A, D608A/D859K, D608K/D859A, D608K/D859K, D608A/D851A/D859A, D608A/D855A/D859A, and D608A/D851A/D855A/D859A mutants in a pDONR vector containing PilY1. Amino acids 532–1163 were cloned out of the pDONR vector for entry into pMCSG7 for protein expression. Vectors were transformed into BL21 Gold (Stratagene) on ampicillin plates overnight and a single colony was used to inoculate a 100 mL LB flask overnight containing 50 µM/mL ampicillin. Cell cultures were centrifuged at 3000×g and the supernatant was discarded. The resultant pellet was used to inoculate a 1.5 L shaker flask of Terrific Broth with 50 µL of antifoam (Sigma-Aldrich) and 50 µM/mL ampicillin. Cells were grown at 37°C until OD_600_ reached 0.6–0.8. Temperature was reduced to 18°C and protein expression was induced with 0.5 mM IPTG. Cells were grown overnight and harvested by centrifugation 6000×g at for 15 minutes at 4°C, and pellets were stored at −80°C.

Cells pellets were thawed using buffer consisting of 25 mM HEPES pH 7.5, 150 mM NaCl, 10 mM imidazole, 5% glycerol, DNase and protease inhibitor tablets (Roche). Cells were sonicated and cell lysate was separated into soluble and insoluble fractions using high-speed centrifugation. The soluble fraction was filtered then nickel purified, buffer exchanged, and separated using an S200 gel filtration column on an ÄKTAxpress™ (GE HealthCare). If necessary, protein and storage buffers were chelated by Chelex-100 to remove bound calcium (Bio-Rad Laboratories). Purified proteins were concentrated to ∼100 µM, frozen, and stored at −80°C.

### Integrin binding assay

Costar EIA/RIA stripwell high binding plates were coated with either 2 µg of purified αVβ5 integrin or 2 µg of purified αVβ3 integrin (Millipore) for the experimental wells, or the molar equivalent of BSA for the control wells, at 100 µL in 100 mM sodium bicarbonate buffer pH 9.6 (Abcam) for 16 hours at 4°C. Plates were washed 1× with 300 µL of PBS. Wells were then blocked with 1% BSA in PBS for one hour at room temperature. Plates were washed 1× with PBS and 100 µg/mL of PilY1 (Wild Type, D621A, Δ619–621, D608A, D608K, D859A, D859K, D608A/D859A, D608A/D859K, D608K/D859A, D608K/D859K, D608A/D851A/D859A, D608A/D855A/D859A, or D608A/D851A/D855A/D859A) in binding buffer (50 mM Tris pH 7.8, 100 mM NaCl 2 mM CaCl_2_, 1 mM MgCl_2_, 1 mM MnCl_2_, and 0.1% Triton) was added to each well including the BSA control wells for 6 hours at 16°C with or without RGDS peptide (inhibitor), GRADSP peptide (control), or no peptide. Metals are needed in the binding buffer for integrin structural integrity. Plates were then washed 3× with PBST (0.1% Tween-20) and 3× with PBS before adding rabbit αPilY1 polyclonal antibody 1∶500 per well in 1% BSA in PBS overnight for 16 hours at 4°C. Plates were then washed 3 times with PBST and 3× with PBS before adding the Alexa fluor 488 f(ab′)_2_ fragment goat anti-rabbit (Invitrogen) 1∶5000 in 1% BSA in PBS protected from light at room temperature for 1 hour. Plates were then washed 3× with PBST, 3× with PBS, then dried. Plates were read on a PHERAstar (BMG LabTech). The experimental and control well averages and standard error of the mean (SEM) were calculated, and then the control average was subtracted from the experimental values and the errors were compounded using the equation √((*experimental SEM*)*^2^*+*(control SEM)*)*^2^*.

### Calcium binding assay

A binding curve for Oregon Green® 488 BAPTA-5N, hexapotassium salt (Invitrogen) in 25 mM HEPES pH 7.5, 250 mM NaCl, 10 mM imidazole, 5% glycerol was measured on a PHERAstar (BMGLabtech) at 488 nm. 20 µM Oregon Green, either 2 µM CaCl_2_, 2 µM MnCl_2_, or 2 µM ZnCl_2_, and PilY1 constructs were serial diluted 1.5–3 fold from ∼100 µM to ∼1 nM to obtain an EC50 which was then used to calculate the respective PilY1 K_d_ for calcium as described previously or PilY1 concentration was held constant and MgCl_2_ concentration was titrated to determine if PilY1 had an affinity to magnesium [23].

### Circular dichroism and thermal denaturation

Protein samples were buffer exchanged into chelated 10 mM K_x_H_x_PO_4_ 50 mM NaF pH 7.7 buffer and brought to 5 µM with or without the addition of CaCl_2_. A wavelength scan from 200–260 λ was performed on a Circular Dichroism Spectrometer 62 DS (Aviv) at 16°C with a 10 second averaging time. Melting temperatures were measures at λ 214 from 3°C to 95°C at one degree increments with a 10 second averaging time.

## Supporting Information

Figure S1 **Circular dichroism for calcium binding mutants.** A–E Molar ellipticity values were calculated for the respective wavelength scans and compared.
(TIFF)

Figure S2 **PilY1 mutation binding curves.** (a) ΔRGD and R619A were modeled to one-site binding. (b) Double calcium binding site mutants D608A/D859K, D608K/D859A, and D608K/D859K. Error represents standard error of the mean.
(TIFF)

Figure S3 **PilY1 alternate metal binding curves.** (a) Oregon Green binding curves were made by titrating magnesium chloride, manganese chloride, or zinc chloride. Curves were fit to one-site saturation (magnesium chloride and manganese chloride) to determine K_d_. (b,c) Zinc and mangansese curves were fit to one-site binding. (d) Magnesium was titrated against Oregon Green, calcium, and PilY1 at the K_d_ to determine affinity.
(TIFF)

Figure S4 **Mutational effects on PilY1:Integrin binding.** As in Figure 2b–d, Wild type PilY1 was established as a reference point and D608A/D851A/D859A, D608A/D855A/D859A, and D608A/D851A/D855A/D859A were measured for integrin binding.
(TIFF)

Table S1 ***P. aeruginosa*** **PilY1 is homologous to PilC family of bacterial adhesin and pilus biogenesis proteins.** PilY1 was individually aligned to *K. Kingae* (Kk) PilC1 and PilC2, *N. gonorrhea* (Ng) PilC1 and PilC2, and *N. meningitidis* (Nm) PilC1 and PilC2 using http://blast.ncbi.nlm.nih.gov/. C-terminal homology indicates residues that are similar (e.g. leucine and isoleucine) while c-terminal identity corresponds to identical residues. Strains used for each protein are as follows: ACF19883 for *Kingella kingae* PilC1, ACF19886.1 for *Kingella kingae* PilC2, O05924_NEIME for *Neisseria meningitidis* PilC1 O05925_NEIME for *Neisseria meningitidis* PilC2, O05923_NEIGO for *Neisseria gonorrhea* PilC1, and Q51019_NEIGO for *Neisseria gonorrhea* PilC2.
(TIFF)

Table S2 **Melting temperatures (T_M_) for mutations of PilY1.** Proteins were scanned at 214 nm after an initial CD scan. T_M_s were calculated using the standard three parameter sigmoidal fit.
(TIFF)
