# Supplementary figures and images for: Pseudomonas aeruginosa PilY1 Binds Integrin in an RGD- and Calcium-Dependent Manner
Source: PLoS One. 2011 Dec 29;6(12):e29629. doi: 10.1371/journal.pone.0029629 (PMC3248442; doi:10.1371/journal.pone.0029629)

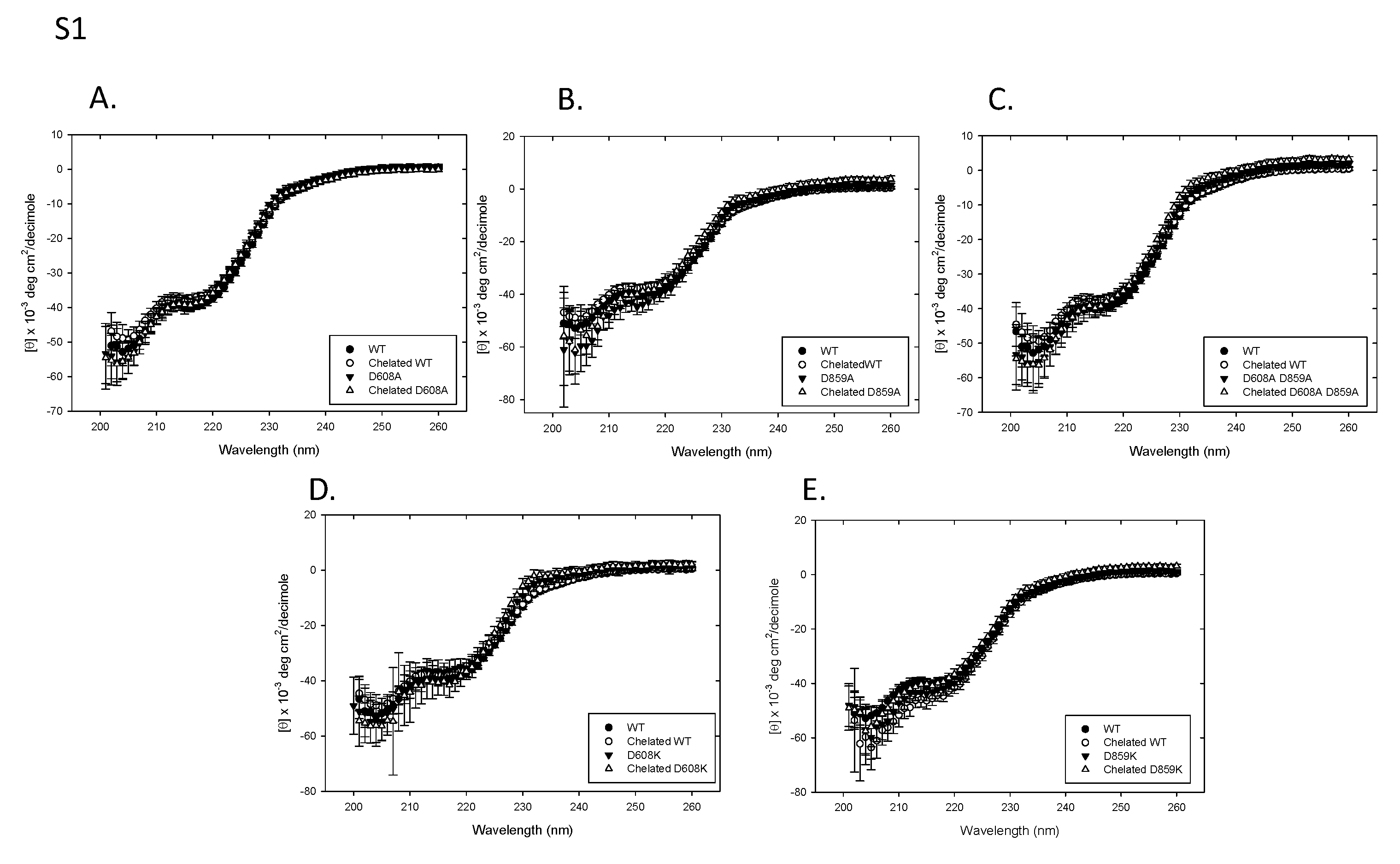

Supplement: Figure S1 — Circular dichroism for calcium binding mutants. A–E Molar ellipticity values were calculated for the respective wavelength scans and compared. (TIFF) [file pone.0029629.s001.tif]

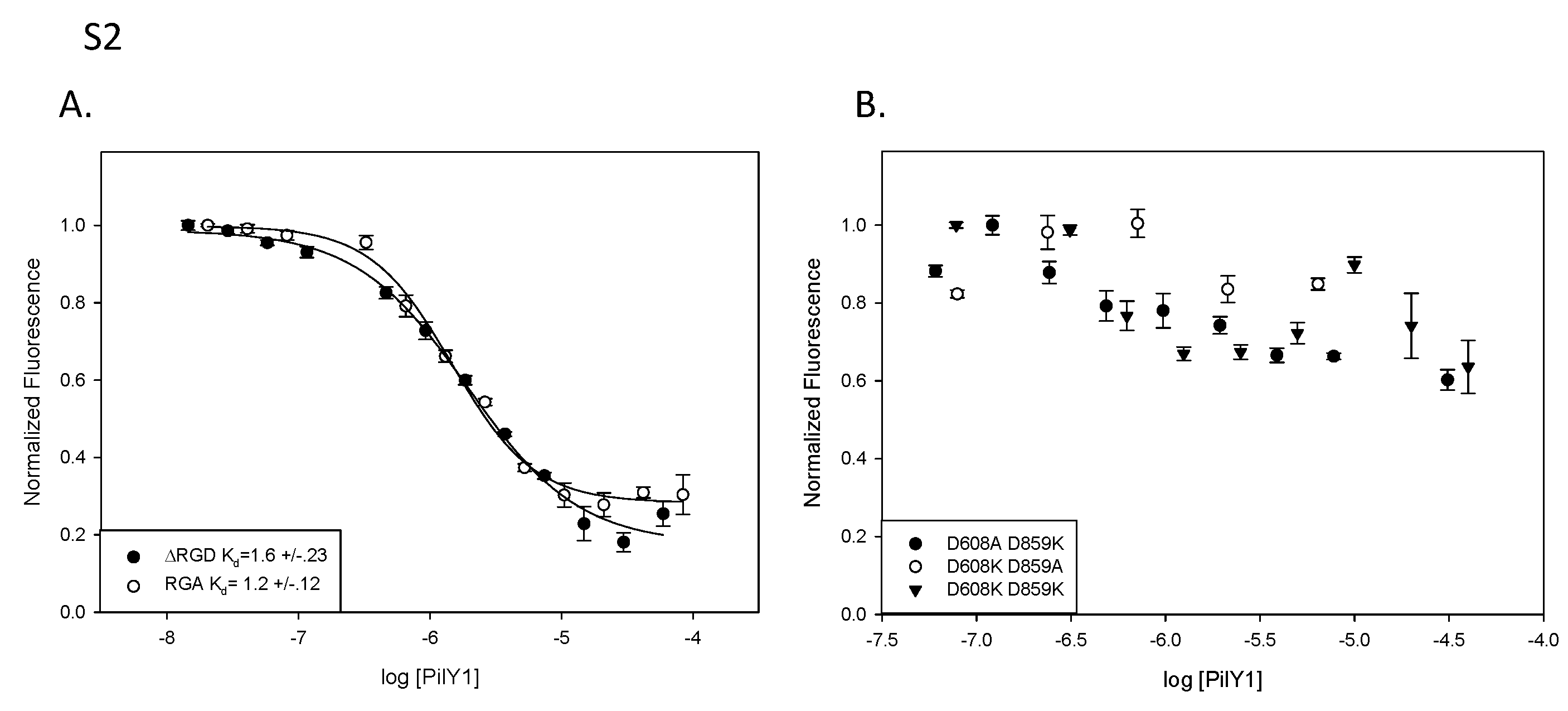

Supplement: Figure S2 — PilY1 mutation binding curves. (a) ΔRGD and R619A were modeled to one-site binding. (b) Double calcium binding site mutants D608A/D859K, D608K/D859A, and D608K/D859K. Error represents standard error of the mean. (TIFF) [file pone.0029629.s002.tif]

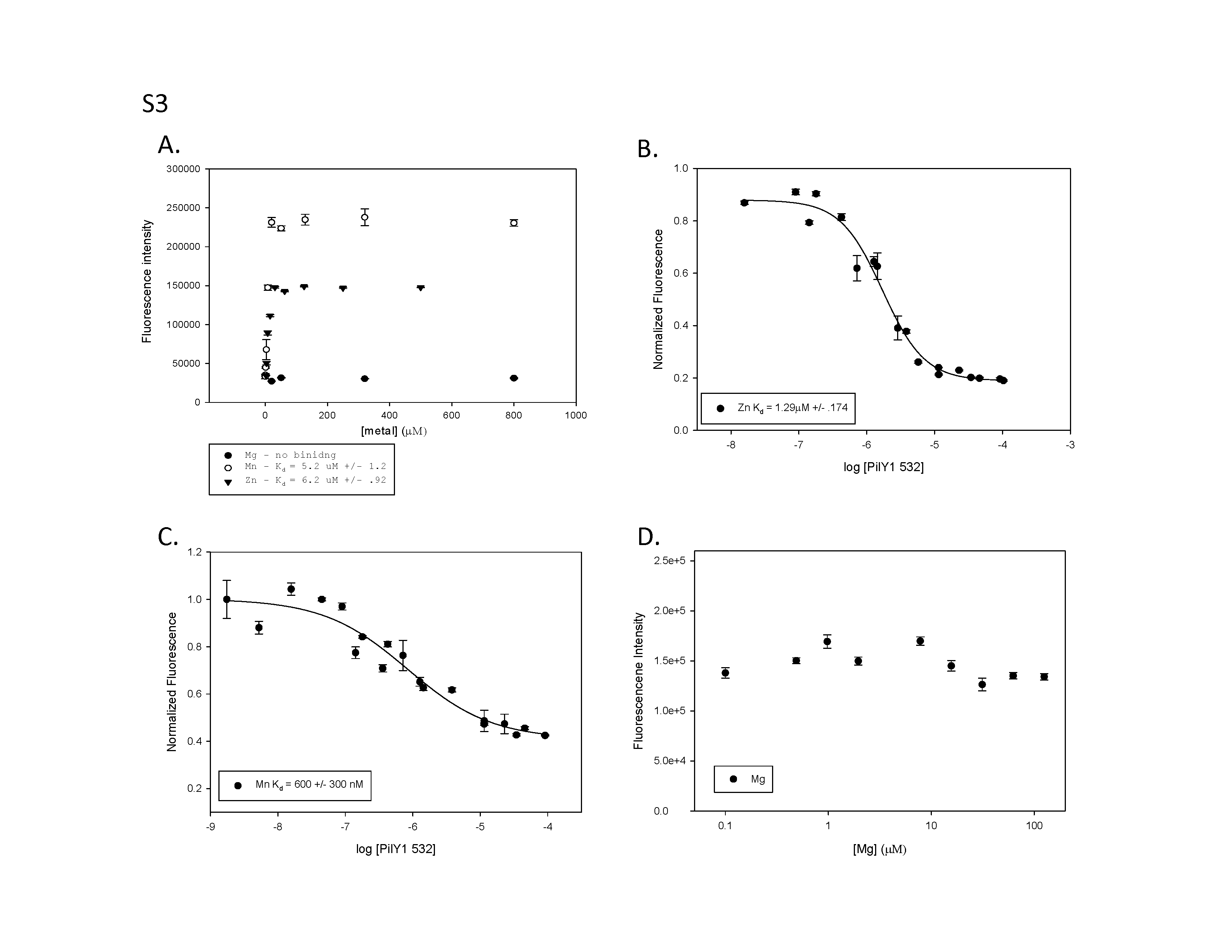

Supplement: Figure S3 — PilY1 alternate metal binding curves. (a) Oregon Green binding curves were made by titrating magnesium chloride, manganese chloride, or zinc chloride. Curves were fit to one-site saturation (magnesium chloride and manganese chloride) to determine Kd. (b,c) Zinc and mangansese curves were fit to one-site binding. (d) Magnesium was titrated against Oregon Green, calcium, and PilY1 at the Kd to determine affinity. (TIFF) [file pone.0029629.s003.tif]

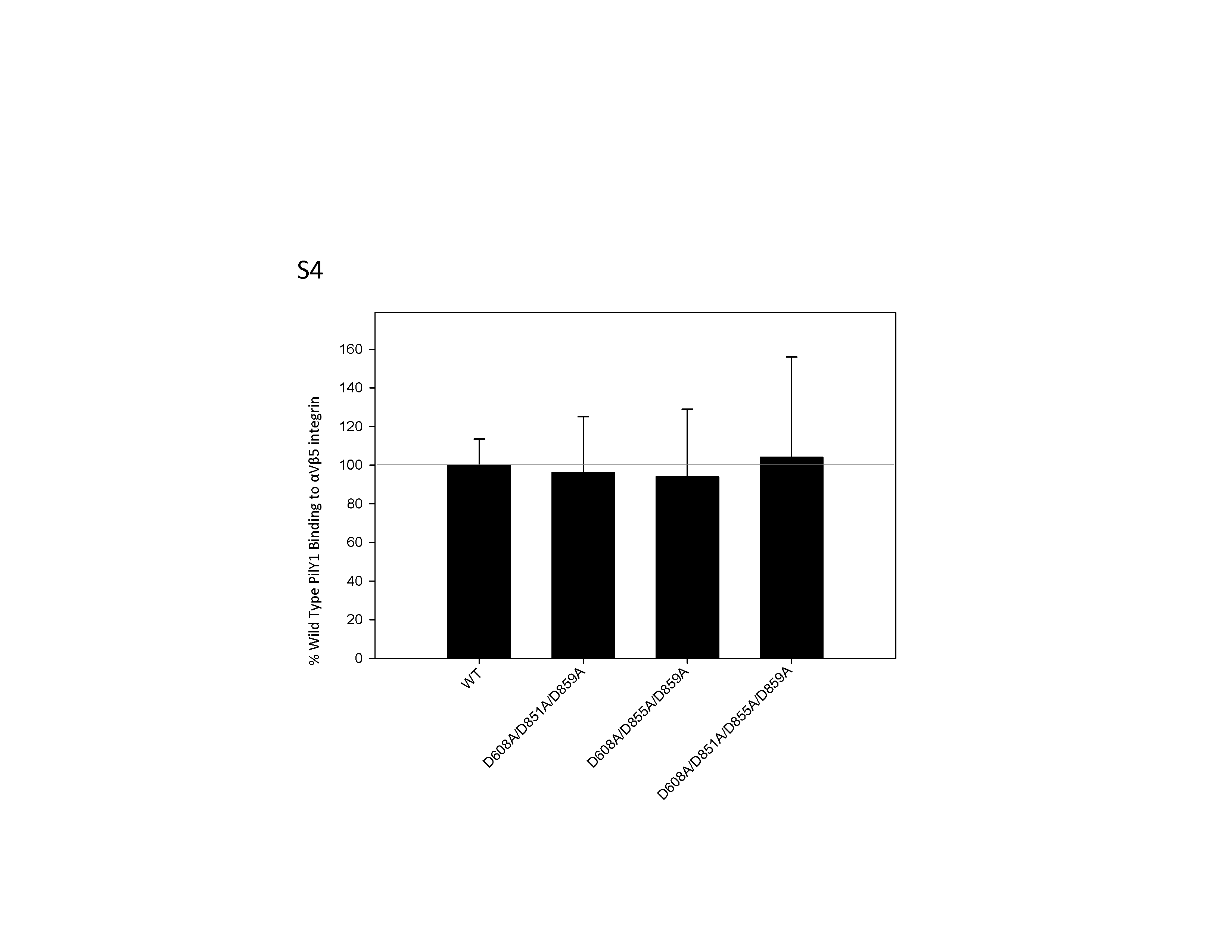

Supplement: Figure S4 — Mutational effects on PilY1:Integrin binding. As in Figure 2b–d, Wild type PilY1 was established as a reference point and D608A/D851A/D859A, D608A/D855A/D859A, and D608A/D851A/D855A/D859A were measured for integrin binding. (TIFF) [file pone.0029629.s004.tif]

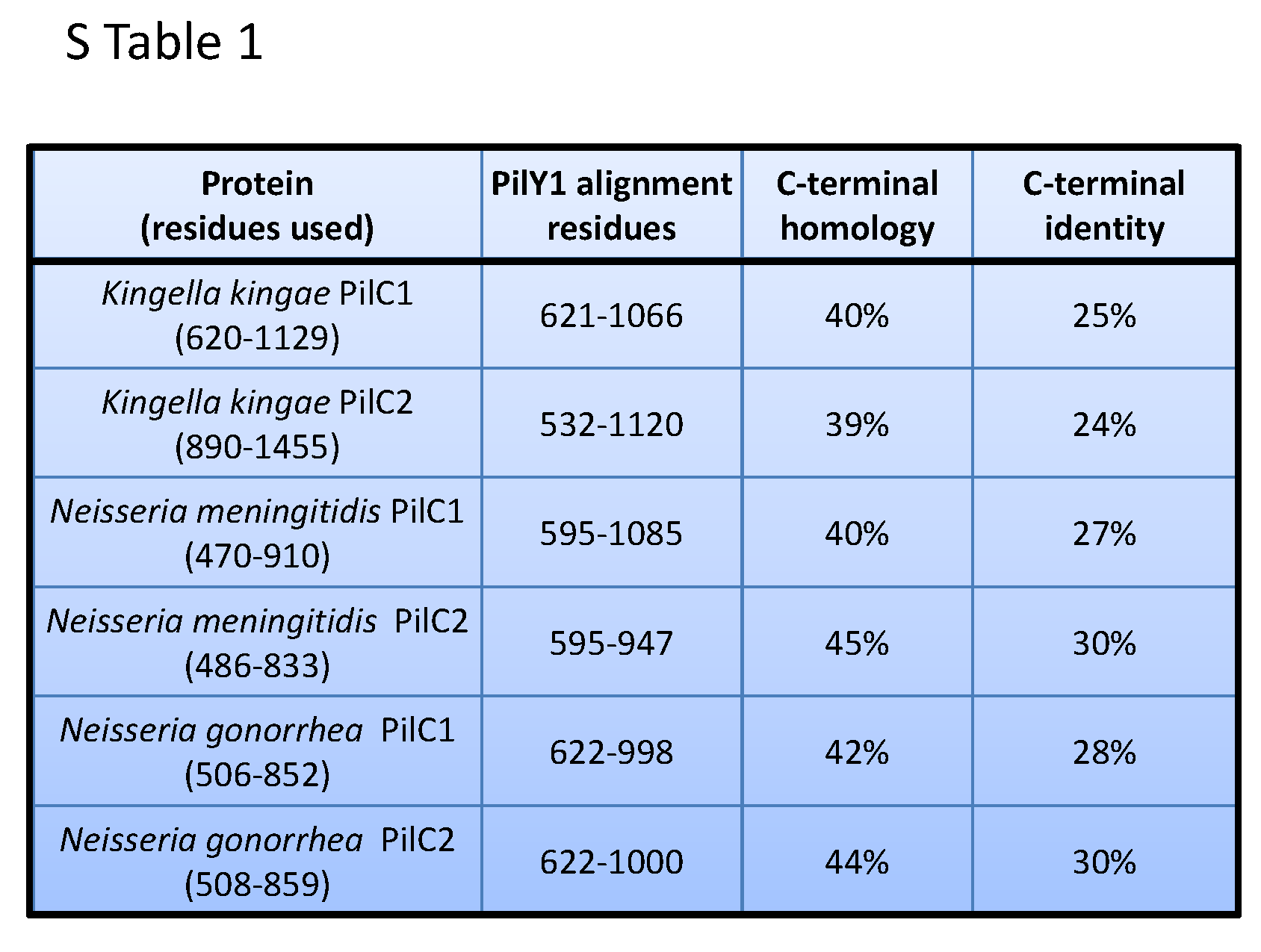

Supplement: Table S1 — P. aeruginosa PilY1 is homologous to PilC family of bacterial adhesin and pilus biogenesis proteins. PilY1 was individually aligned to K. Kingae (Kk) PilC1 and PilC2, N. gonorrhea (Ng) PilC1 and PilC2, and N. meningitidis (Nm) PilC1 and PilC2 using http://blast.ncbi.nlm.nih.gov/. C-terminal homology indicates residues that are similar (e.g. leucine and isoleucine) while c-terminal identity corresponds to identical residues. Strains used for each protein are as follows: ACF19883 for Kingella kingae PilC1, ACF19886.1 for Kingella kingae PilC2, O05924_NEIME for Neisseria meningitidis PilC1 O05925_NEIME for Neisseria meningitidis PilC2, O05923_NEIGO for Neisseria gonorrhea PilC1, and Q51019_NEIGO for Neisseria gonorrhea PilC2. (TIFF) [file pone.0029629.s005.tif]

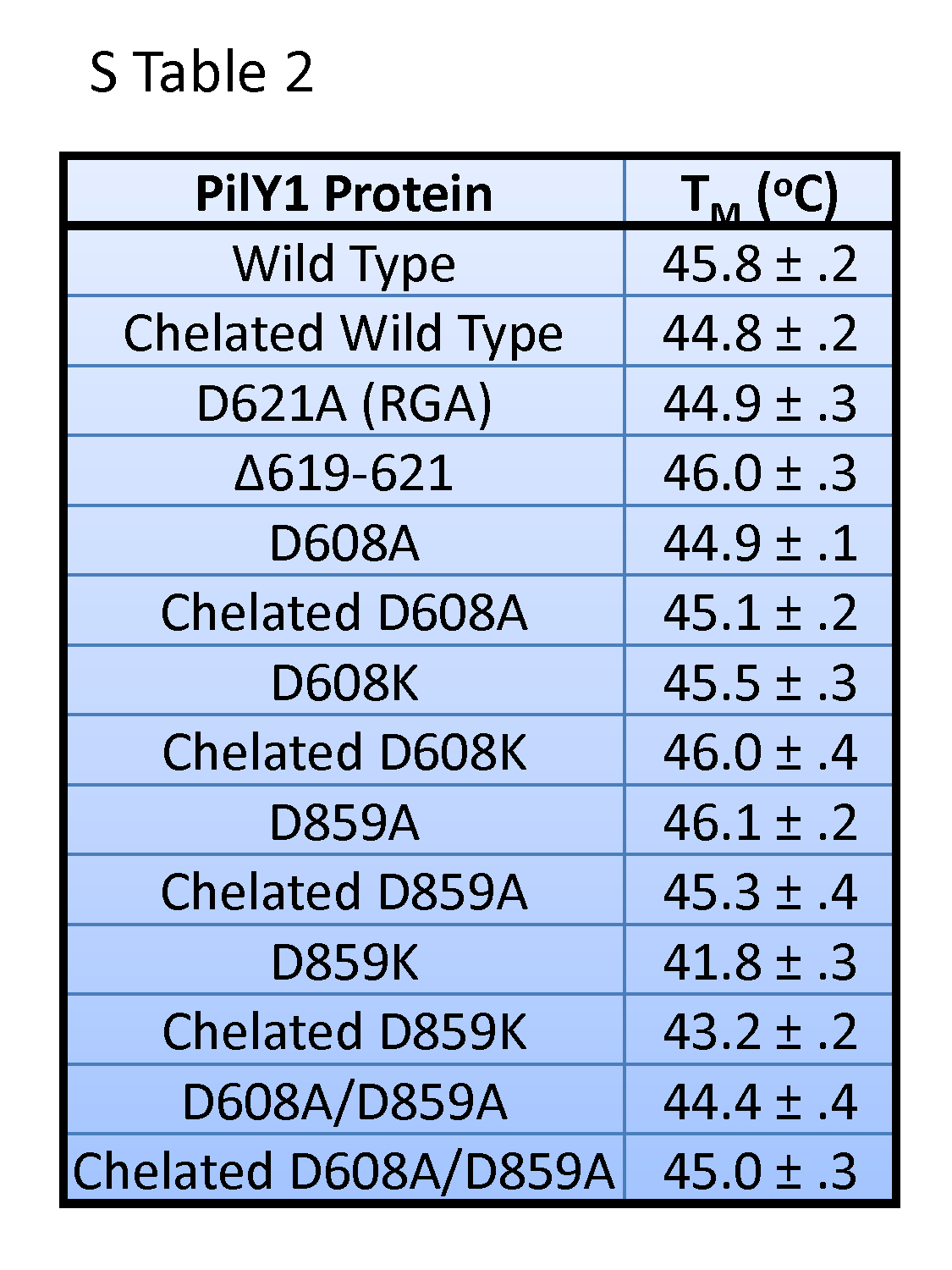

Supplement: Table S2 — Melting temperatures (TM) for mutations of PilY1. Proteins were scanned at 214 nm after an initial CD scan. TMs were calculated using the standard three parameter sigmoidal fit. (TIFF) [file pone.0029629.s006.tif]
